# Assessing knowledge of healthcare providers concerning cardiovascular risk after hypertensive disorders of pregnancy: an Australian national survey

**DOI:** 10.1186/s12884-020-03418-5

**Published:** 2020-11-23

**Authors:** Heike Roth, Caroline S. E. Homer, Clare Arnott, Lynne Roberts, Mark Brown, Amanda Henry

**Affiliations:** 1 Faculty of Health, University of Technology Sydney, Sydney, NSW Australia; 2 Burnet Institute, Maternal and Child Health, Melbourne, Victoria Australia; 3 The George Institute, Sydney, NSW Australia; 4 Department of Cardiology, RPA, Sydney, NSW Australia; 5 St George Hospital, Sydney, NSW Australia; 6 St George & Sutherland Clinical School, UNSW, Sydney, NSW Australia; 7 School of Women’s and Children’s Health, UNSW Medicine, University of NSW, Sydney, NSW Australia

**Keywords:** Cardiovascular risk, Healthcare providers, Preeclampsia, Gestational hypertension, Longterm cardiovascular health, Preventive health

## Abstract

**Background:**

Hypertensive disorders of pregnancy (HDP) affect 5–10% of pregnant women. Women after HDP have 2–3 times increased risk of heart attack, stroke and diabetes, as soon as 5–10 years after pregnancy. Australian healthcare providers’ knowledge of cardiovascular disease (CVD) risks for women after HDP is unknown, and this study aimed to explore their current knowledge and practice regarding long-term cardiovascular health after HDP, as a precursor to producing targeted healthcare provider education on health after HDP.

**Methods:**

A custom-created, face-validated online survey explored knowledge about long-term risks after HDP. Distribution occurred from February to July 2019 via professional colleges, key organisations and social media. The objective was to assess current knowledge and knowledge gaps amongst a group of healthcare providers (HCP) in Australia, regarding long-term cardiovascular health after hypertensive disorders of pregnancy (HDP), specifically gestational hypertension or preeclampsia.

**Results:**

Of 492 respondents, 203 were midwives, 188 obstetricians, 75 general practitioners (GP), and 26 cardiologists. A risk knowledge score was computed with 0–6 considered low, 6.1–8.9 moderate and 9–12 high. Most participants (85%) were aware of increased cardiovascular disease after preeclampsia and gestational hypertension (range 76% midwives to 100% cardiologists). There were significant differences in average knowledge scores regarding health after preeclampsia; high for cardiologists (9.3), moderate for GPs and obstetricians (8.2 and 7.6 respectively) and low for midwives (5.9). Average knowledge scores were somewhat lower for gestational hypertension (9.0 for cardiologists, 7.4 for obstetricians and GPs, 5.1 for midwives). Knowledge was highest regarding risk of chronic hypertension, moderate to high regarding risk of ischaemic heart disease, stroke and recurring HDP, and low for diabetes and peripheral vascular disease. Only 34% were aware that risks start < 10 years after the affected pregnancy.

**Conclusion(s):**

Participants were aware there is increased cardiovascular risk after HDP, although less aware of risks after gestational hypertension and some specific risks including diabetes. Findings will inform the development of targeted education.

**Supplementary Information:**

The online version contains supplementary material available at 10.1186/s12884-020-03418-5.

## Background

Hypertensive disorders of pregnancy (HDP) includes preeclampsia (PE), gestational hypertension (GH) and pre-existing or chronic hypertension (CH) and complicates 5–10% of pregnancies [1]. PE is a multi-system disorder, characterised by hypertension and involvement of one or more other organ systems and/or the fetus [2, 3]. Gestational hypertension is new onset hypertension without any other complications during pregnancy and has little association with adverse pregnancy outcomes apart from increased risk of progression to preeclampsia [2, 3]. Both conditions are associated with long-term cardiovascular sequelae [4, 5]. Cardiovascular disease (CVD), the leading cause of death in women globally [6], is up to two and a half times higher for women after HDP versus those with no HDP [4, 5, 7, 8]. This increased risk remains after adjusting for the presence of other cardiovascular risk factors and is present within 5–10 years after the affected pregnancy [8–12].

Both Australian and international societies, including the Society of Obstetric Medicine of Australia and New Zealand (SOMANZ) and the International Society for the Study of Hypertension in Pregnancy (ISSHP), recommend women and healthcare providers (HCP) are provided with information about HDP and later CVD, and HCPs should ask women about their HDP history when assessing cardiovascular health and risk factors. This includes review at 3 months postpartum and regular follow-up with a GP to monitor blood pressure, fasting lipids and blood sugar [2]. Recommendations emphasise adoption of a healthy lifestyle with maintenance of an ideal weight and regular aerobic exercise [2, 3]. Despite existing evidence and recommendations, it is unknown whether Australian HCPs are aware of the association between HDP and CVD [13]. The aim of this study was to explore Australian HCPs current knowledge and practice regarding long-term cardiovascular health after HDP, as a precursor to producing targeted HCP education on health after HDP.

## Method

A national, multidisciplinary survey of HCPs was conducted, using a custom-created, face-validated online survey (Additional file 1). Ethical approval was provided by South-Eastern Sydney Human Research Ethics Committee (Ref: 18/POWH/326).

### Face validation of the survey

As a validated instrument to assess HCP’s knowledge and practice was unavailable, a survey was custom designed. The survey was initially compiled from a scoping literature review [13] and complemented by questions specifically exploring the Australian context. Twenty-one HCPs across eight professions (obstetricians, cardiologists, nephrologists, obstetric physicians, anaesthetists, general practitioners, midwives and community health nurses) participated in the face-validation process. These HCPs commented on content, language, flow, survey structure including length, whether the risk profile at survey conclusion was informative, and potential value of the survey data. The survey was modified until consensus over a final version was achieved.

### Data collection

The online survey, powered by SurveyMonkey (Survey Monkey Inc., San Mateo, USA), was open from 15 February until 4 August 2019. Survey distribution occurred through professional organisations, namely: The Royal Australian and New Zealand College of Obstetricians and Gynaecologists (RANZCOG) [targeted to DRANZCOG holders (General Practitioners with obstetrics and gynaecology diploma) and FRANZCOG (Fellow) members], the Australian College of Midwives (ACM), and the Cardiac Society of Australia and New Zealand (CSANZ). Additionally, distribution occurred via the study team’s professional networks, as well as social media pathways such as Twitter and Facebook. The targeting of general practitioners/family doctors (GPs) with an obstetric diploma, and therefore specialised in maternity care and women’s health, was a deliberate decision. With our survey identifying a ‘best-case’ knowledge scenario within this group of HCPs meant that we expected our sample to have higher overall knowledge on this topic, relative to all Australian HCPs in the included professions, setting an upper limit regarding future targeted education.

The survey collected demographic details and assessed HCPs general and specific knowledge of risk after HDP, and their practices around consultation and follow up of women with a history of HDP. Early in the survey, HCPs were asked ‘Do you think that there is an increased risk of developing future cardiovascular disease after gestational hypertension or preeclampsia?’. Those who answered ‘Neither gestational hypertension or preeclampsia increase the long-term health risks’, were sent to the risk profile at the end of the survey so that detailed questions regarding risk were only being asked of those HCPs with some knowledge of CVD and HDP links. The HCPs were asked to classify the risk of women with a history of GH or PE, of various long-term health outcomes as ‘less than’, ‘equal to’ or ‘greater than’ that of a woman with a normotensive pregnancy. The survey included conditions that women are at increased risk of after HDP (chronic hypertension, CVD, diabetes, renal disease) and also those with similar prevalence (breast cancer, leukaemia and seizures). Upon survey completion, HCPs were provided with a correct risk profile summary and a link to further information. Commencement of the survey was taken as consent to participate.

### Data analysis

Quantitative survey analysis was undertaken using SPSS Version 25 (SPSS Statistics for Windows, Armonk, NY). Demographic data and responses to individual questions were analysed descriptively. To examine difference in knowledge levels amongst the targeted HCP subgroups, (obstetricians, GPs, midwives, cardiologists) responses regarding HDP and future health risks were compared using Chi-squared testing for categorical data and one-way ANOVA for continuous data. A *p-*value of < 0.05 was considered statistically significant.

For ease of interpretation, a knowledge score was created for the GH and PE risk matrix, whereby 1 point was allocated to the correct answer, 0 for the incorrect answer, 0 for ‘I do not know’ and 0 for no answer/left blank. A mean score for each risk factor was calculated and a scale of ‘low’, ‘moderate’ and ‘high’ knowledge was established. The ranking classifications were chosen based on the data distribution. For individual risk mean scores, ‘low knowledge’ equated to a mean of 0.00–0.50, ‘moderate knowledge’ was 0.51–0.80 and ‘high knowledge’ a mean of 0.81–1.00. An overall mean score out of 12 (as there were 12 conditions) was given for GH and PE for each profession. A ‘low knowledge’ equated to a mean of 0–6 (50% or less correct answers), a mean of 6.1–8.9 was considered ‘moderate knowledge’, and a mean score of 9–12 was considered ‘high knowledge’.

## Results

**Fig. 1:**
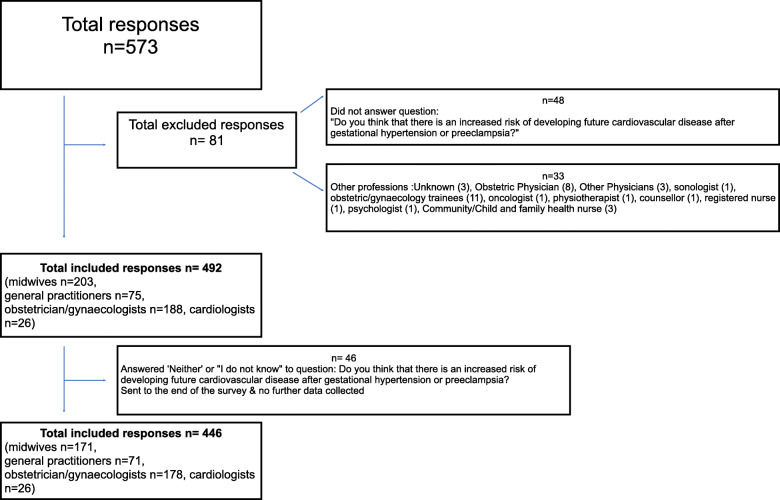
Survey inclusion. Flowchart of respondents included and excluded in the survey analysis

In total, 573 survey responses were received (Fig. 1). Eighty-one were excluded: 48 for not answering the key inclusion question ‘Do you think that there is an increased risk of developing future cardiovascular disease after gestational hypertension or preeclampsia?’ and 33 for representing diverse professions that were not the target HCPs. Of 492 included responses 203 (41%) were from midwives, 75 (15%) GPs, 188 (38%) obstetrician/gynaecologists and 26 (5%) cardiologists. Of these, 446 provided responses to the detailed knowledge questions. Most respondents were female (82%) and approximately half had > 15 years’ experience in their profession, with the exception of cardiologists (70% 10 years or less in the profession). Almost all respondents (94%) see/treat women with a history of PE or GH, and the majority (78%) stated they routinely ask women about their pregnancy history including GH or PE (Table 1). Most respondents were aware of the increased CVD risk after both PE and GH (85%), while 6% thought only PE (4%) or GH (2%) increased risk, but not both (Table 2). The 9.3% who did not know (8.5%) or believed that neither GH nor PE (0.8%) carried a risk were directed to the end of the survey, with the remaining 446 respondents directed towards more in-depth knowledge questions.

**Table 1:** Respondent demographics

	Midwives n(%)	GPs n(%)	Obstetrician n(%)	Cardiologists n(%)	Total n(%)^a^
Total N	**203**	**75**	**188**	**26**	**492**
Sex					
*Female*	200 (98)	63 (84)	119 (63)	22 (85)	404 (82)
*Male*	2 (1)	12 (16)	66 (35)	4 (15)	84 (17)
*Prefer not to answer*	1 (1)	0 (0)	3 (2)	0 (0)	4 (1)
Years of experience					
*< 5 years*	38 (19)	12 (16)	23 (12)	10 (39)	83 (17)
*5–10 years*	28 (14)	17 (23)	47 (25)	8 (31)	100 (24)
*11–15 years*	29 (14)	9 (12)	26 (14)	3 (12)	67 (14)
*> 15 years*	107 (53)	36 (48)	92 (49)	5 (19)	240 (49)
*Prefer not to answer*	1 (0.5)	1 (1)	0 (0)	0 (0)	1 (0)
State of Practice					
*New South Wales*	103 (51)	17 (23)	55 (29)	8 (31)	183 (37)
*Victoria*	29 (14)	23 (31)	39 (21)	17 (65)	108 (22)
*Australian Capital Territory*	7 (4)	0 (0)	8 (4)	0 (0)	15 (3)
*Queensland*	26 (13)	13 (17)	35 (19)	0 (0)	74 (15)
*Northern Territory*	5 (3)	2 (3)	4 (2)	0 (0)	11 (2)
*South Australia*	17 (8)	5 (7)	16 (9)	1 (4)	39 (8)
*Tasmania*	5 (3)	0 (0)	11 (6)	0 (0)	16 (3)
*Western Australia*	9 (5)	15 (20)	20 (11)	0 (0)	44 (9)
See/Treat women with history of PE or GH					
*Yes*	139 (95)	73 (97)	177 (94)	18 (69)	461 (94)
*No*	10 (5)	2 (3)	11 (6)	8 (31)	31 (6)
Routinely ask about pregnancy history including GH or PE					
*Always*	174 (86)	40 (53)	161 (86)	8 (31)	383 (78)
*Often*	18 (9)	21 (28)	16 (8)	8 (31)	63 (13)
*Sometimes*	8 (4)	14 (19)	11 (6)	9 (35)	42 (9)
*Never*	3 (2)	0 (0)	0 (0)	1 (4)	4 (1)

*PE* preeclampsia, *GH* gestational hypertension

^a^ percentages may not add to 100% as figures are rounded to whole numbers only

**Table 2:** Respondent answers on existence of cardiovascular risk after preeclampsia and/or gestational hypertension

	Midwives ***n*** = 203	GPs ***n*** = 75	Obstetricians ***n*** = 188	Cardiologists ***n*** = 26	Total ***n*** = 492
	***n*** = yes (%)	***n*** = yes (%)	***n*** = yes (%)	***n*** = yes (%)	***n*** = yes (%)
*PE only*	12 (6)	1 (1)	7 (4)	0 (0)	20 (4)
*GH only*	4 (2)	3 (4)	2 (1)	0 (0)	9 (2)
*PE and GH*	155 (76)	67 (89)	169 (90)	26 (100)	417 (85)
*Neither*	1 (1)	0 (0)	3 (2)	0 (0)	4 (1)
*I am not sure*	31 (15)	4 (5)	7 (4)	0 (0)	42 (9)
*Proceed to rest of survey*	171 (84)	71 (95)	178 (95)	26 (100)	446 (91)
*Discontinued from data collection*	32 (16)	4 (5)	10 (5)	0 (0)	46 (9)

Overall, most professions had ‘high’ knowledge with regards to women developing chronic hypertension after PE and GH (Table 3). Although ‘high’ knowledge was displayed for HDP recurrence after PE (‘moderate’ for midwives at 0.72, GP 0.84, obstetricians 0.90, cardiologists 0.96), more varied results were noted for recurrence of HDP after GH, ranging from ‘low’ for midwives, ‘moderate’ for GPs and obstetricians to ‘high’ for cardiologists. Lowest knowledge across all four professions regarded future diabetes risk for both PE (range midwives 0.30 to cardiologists 0.81) and GH (from midwives 0.25 to cardiologists 0.65). Another low scoring condition was peripheral vascular disease (PVD), where knowledge was ‘low’ to ‘moderate’ for both PE and GH. Additional file 2 shows the detailed breakdown of respondent answers, including proportion answering ‘I don’t know’ or skipping questions versus giving a firm but incorrect answer. Conditions with the highest proportion of “I don’t know” answers were diabetes, PVD, and the three distractors (breast cancer, leukaemia and seizures).

**Table 3:** Means of risk factor knowledge score by profession and by pregnancy HDP (PE or GH)

	Midwives ***n*** = 171		GPs ***n*** = 71		Obstetricians ***n*** = 178		Cardiologists ***n*** = 26		***P***	
	PE	GH	PE	GH	PE	GH	PE	GH	PE	GH
*CH*	0.70 (mod)	0.65 (mod)	0.83 (high)	0.85 (high)	0.88 (high)	0.86 (high)	1.00 (high)	1.00 (high)	≤0.001	≤0.001
*Diabetes*	0.30 (low)	0.25 (low)	0.39 (low)	0.41 (low)	0.40 (low)	0.39 (low)	0.81 (high)	0.65 (mod)	≤0.001	≤0.001
*Renal Disease*	0.63 (mod)	0.50 (low)	0.81 (high)	0.71 (mod)	0.81 (high)	0.71 (mod)	1.00 (high)	0.88 (high)	≤0.001	≤0.001
*Cardiac Death*	0.54 (mod)	0.46 (low)	0.76 (mod)	0.67 (mod)	0.79 (mod)	0.69 (mod)	0.88 (high)	0.81 (high)	≤0.001	≤0.001
*IHD/MI*	0.56 (mod)	0.48 (low)	0.76 (mod)	0.77 (mod)	0.82 (high)	0.74 (mod)	0.96 (high)	0.92 (high)	≤0.001	≤0.001
*HDP repeat*	0.72 (mod)	0.49 (low)	0.84 (high)	0.77 (mod)	0.90 (high)	0.74 (mod)	0.96 (high)	0.92 (high)	≤0.001	≤0.001
*Stroke*	0.60 (mod)	0.52 (mod)	0.76 (mod)	0.72 (mod)	0.80 (mod)	0.68 (mod)	0.92 (high)	0.88 (high)	≤0.001	≤0.001
*PVD*	0.47 (low)	0.41 (low)	0.59 (mod)	0.51 (mod)	0.58 (mod)	0.54 (mod)	0.73 (mod)	0.65 (mod)	0.250	0.022
*Overall Mortality*	0.61 (mod)	0.51 (mod)	0.77 (mod)	0.67 (mod)	0.78 (mod)	0.71 (mod)	0.92 (high)	0.88 (high)	≤0.001	≤0.001
*Breast Cancer*^a^	0.28 (low)	0.28 (low)	0.43 (low)	0.41 (low)	0.48 (low)	0.46 (low)	0.42 (low)	0.50 (low)	≤0.001	≤0.001
*Leukaemia*^a^	0.29 (low)	0.28 (low)	0.32 (low)	0.37 (low)	0.46 (low)	0.46 (low)	0.46 (low)	0.50 (low)	0.003	≤0.001
*Seizures*^a^	0.16 (low)	0.25 (low)	0.33 (low)	0.49 (low)	0.44 (low)	0.46 (low)	0.27 (low)	0.35 (low)	≤0.001	≤0.001
***Overall mean Knowledge score (out of 12)***	**5.85** (low)	**5.08** (low)	**7.59** (mod)	**7.36** (mod)	**8.15** (mod)	**7.45** (mod)	**9.35** (high)	**8.96** (high)	***≤*0.001**	***≤*0.001**

*CH* chronic hypertension, *IHD* ischaemic heart disease, *MI* myocardial infarction, *HDP* hypertensive disorder of pregnancy, *PVD* peripheral vascular disease, *mod* moderate

^a^ Breast cancer, leukaemia and seizures are distractors within the survey. These were included despite being conditions that women after HDP are not at greater risk of

Overall average knowledge scores were ‘low’ for midwives (5.9 for PE and 5.1 for GH), ‘moderate’ for GPs (7.6 PE, 7.4 GH) and obstetricians (8.2 PE, 7.5 GH) and ‘high’ for cardiologists (9.3 PE and 9.0 GH). Only 34% were aware that the risks start to manifest less than 10 years after an affected pregnancy (Table 4). Regarding usual practice around risk discussion with women after HDP, the most frequent practices by all professions were assessing CVD risk (61%) and recommending lifestyle changes (66%) (Table 5).

**Table 4:** Respondent answers concerning timing of risk signs and symptoms rise after HDP in numbers and percentages

	Midwives ***n*** = 171	GPs ***n*** = 71	Obstetricians ***n*** = 178	Cardiologists ***n*** = 26	Total ***n*** = 446
	n (%)^a^	n (%)^a^	n (%)^a^	n (%)^a^	n (%)^a^
*< 10 years after pregnancy*	44 (28)	25 (38)	67 (39)	14 (54)	150 (34)
*11–15 years after pregnancy*	46 (29)	29 (44)	71 (42)	10 (39)	156 (32)
*16–20 years after pregnancy*	17 (11)	5 (8)	9 (5)	1 (4)	32 (7)
*> 20 years after pregnancy*	11 (7)	1 (2)	6 (4)	0 (0)	12 (3)
*Not sure/I don’t know*	42 (26)	6 (9)	17 (10)	1 (4)	49 (11)
*Did not answer*	11 (6)	5 (7)	8 (4)	0 (0)	24 (5)
*Total answers*	160 (93)	66 (93)	170 (96)	26 (100)	422 (95)

^a^ Percentages may not add to 100% as figures are rounded to whole numbers only

**Table 5:** Healthcare provider’s practice regarding risk discussions in numbers and percentages

	Midwives ***n*** = 171	GPs ***n*** = 71	Obstetricians ***n*** = 178	Cardiologists ***n*** = 26	Total ***n*** = 446
	n (%)^b^	n (%)^b^	n (%)^b^	n (%)^b^	n (%)^b^
*Assess CV risk*	69 (40)	59 (83)	120 (67)	23 (88)	271 (61)
*Medication*	21 (12)	16 (23)	28 (16)	10 (38)	75 (17)
*Lifestyle adjustments*	84 (49)	56 (79)	134 (75)	20 (77)	294 (66)
*Not discussed risk*	68 (40)	4 (6)	15 (8)	2 (8)	89 (20)
*Other*^a^	4 (2)	0 (0)	3 (2)	0 (0)	7 (2)

^a^ Other includes (*n* = 7): Further specialist follow up (*n* = 3), referral letter to GP informing of risks and organise long-term care post HDP (*n* = 1), mental health assessment/solutions (*n* = 2), advise women to disclose HDP as part of their medical history (*n* = 1)

^b^Percentages may not always add up to 100% as HCPs were asked to select any/all that apply

## Discussion

In this novel Australian HCP survey, most respondents (85%) were aware that both GH and PE increase the long-term risk of CVD and ‘always’ (78%) ask about HDP history. Despite this reasonably high awareness of HDP being linked to future CVD risks, we identified significant knowledge gaps regarding individual conditions. All professions had consistently lower knowledge scores regarding conditions after GH. This may be because GH is seen as a more benign form of HDP, although studies show GH has similar CVD risk to preeclampsia [4, 14].

Within the context of the selected sample of HCPs, findings were expected to reflect a ‘best-case’ scenario of knowledge as their specialised training theoretically indicates high overall knowledge. Of the total respondent number (*n* = 573), 48 (8%) were excluded for not answering the key risk question asked (‘Do you think that there is an increased risk of developing future cardiovascular disease after gestational hypertension or preeclampsia?’) and 46 of the *n* = 492 respondents (9%) for being unsure or not believing there are health risks after HDP. Therefore, even in this sample, with sufficient interest in the topic to undertake the survey, a minority had very low or incorrect overall knowledge. Education developed will need to cater to HCPs with no pre-existing knowledge as well as focus on the specific gaps identified by the survey.

International studies exploring HCPs knowledge have reported overall low knowledge [13]. These studies feature results from highly specialised HCPs with substantial involvement in maternity and women’s health care. Only one study has examined knowledge of HCPs on long-term health risks after both PE and GH, whilst all others focus on risks after PE only [15]. In line with another study that found that higher knowledge was associated with belonging to a particular profession [16], we found higher knowledge among medical professionals compared with midwives. However, in contrast to an American study that found obstetricians generally had more awareness of CVD after PE than internal medicine physicians [17], cardiologists were the highest scoring profession in this survey, DRANZCOG GPs and obstetricians were quite similar.

This study identified some significant knowledge gaps amongst specialist HCPs. Our study findings resonate with those from similarly targeted HCPs in Canada, Germany, Nigeria and the USA conducted between 2007 and 2017 [13]. Therefore, from a global perspective, this reinforces the research to practice gap in yet another country a few years on. With international guidelines, including ISSHP 2018 [2], specifically targeted to assist HCPs on an international scale to better manage and address health after HDP, this practice gap would be expected to narrow.

Given the different scope of practice of various professions, different knowledge and knowledge gaps were expected, and our results can help tailor future education of different HCPs on this topic. For example, options might include improving knowledge about the risks associated with GH amongst specialist GPs and obstetricians. Once this educational material has been piloted with the specialised HCPs, it may be adapted to suit a broader distribution which would include, for example, GPs without specialist qualification in women’s health.

In this study, the condition following PE or GH associated with the highest knowledge was chronic hypertension, consistent with previous HCP studies [15–18]. Knowledge was lowest with regards to PVD and diabetes across all groups. The wide range of knowledge levels displayed within this study concerning risk of recurring HDP was an unexpected finding and suggests further need for maternity care provider education on this topic.

Only one-third of respondents were aware that risks start to manifest under 10 years after an HDP pregnancy, which may negatively impact on timely follow up and counselling of affected women. In combination with predominantly low to moderate knowledge of most individual CVD conditions explored within this study, this suggests opportunities are currently being lost to discuss preventive strategies that could improve women’s health trajectories. The majority of participants were female. Given that midwifery is a predominantly female occupation in Australia, and GPs and obstetricians closer to 50:50, the response rate of male versus female within these three professions is not unexpected. However, given that a minority of Australian cardiologists are female, the high fraction of cardiology respondents being women suggests bias in this sample.

As with all surveys, it is uncertain how representative it is of the population under study i.e. it is unknown whether knowledge of non-respondents is comparable to that of respondents. Furthermore, the number of respondents in all included subgroups are a small proportion of the national registers (particularly cardiologists) which suggests volunteer bias and also affects generalisability. However, non-representative national HCP numbers along with a highly specialised sample of HCPs can be noted within all research addressing HCP knowledge [13]. This study was also subject to sample limitations as specialised maternity and women’s health HCPs with prior knowledge of the link between HDP and CVD were included in the analysis e.g. the targeting of GP distribution to DRANZCOG holders. This was, however, a deliberate decision, since it can reasonably be expected that these specialised GPs have highest, relative knowledge. Therefore, the knowledge gaps that were found can be expected to extend to the wider Australian GP population. In addition, we targeted these specialised GPs with awareness that response rates to GP surveys are generally very low. For example, recruited numbers were < 15% in this study despite various, targeted recruitment strategies in place [19]. A more general/inclusive spread of midwives, GPs, obstetricians would likely have lesser knowledge than our sample as fewer maternal health qualifications (GPs) and/or not be interested enough in the topic to take the survey. Therefore, when designing education it would be wise to cater for no higher than the levels of knowledge exhibited in our sample, and also cater for lower levels of knowledge.

Our custom-created knowledge score is both a strength, as it allows for a summary of findings across all the conditions and risks, and a limitation, as assigning cut-points is an arbitrary designation. Having included the distractor conditions (breast cancer, leukaemia and seizures) may also have altered the overall score. However, we believe it is important that knowledge is both of conditions that actually do occur more often after GH/PE, plus not incorrectly believing these women are at increased risk of more conditions than they are.

### What are the implications?

Research on increased CVD risk after HDP emerged in the early 2000s with the first systematic review published in 2007 [20]. Since then, further research has supported these findings [7], providing close to two decades worth of data signalling the link between HDP and increased CVD risk. Given the length of time that this topic has been addressed in research, it can be an expectation that this knowledge would by now have been translated into practice, particularly amongst our sample that was most likely to include ‘best-case knowledge’ HCPs. That our results did not find this suggests both an even greater knowledge gap in those unaware of the link as well as amongst the non-specialised groups, and ongoing failure to close the knowledge to practice gap on health after HDP. Therefore, this study is valuable from the public health perspective, given the wider context of prevalence and importance of cardiovascular disease in women.

ISSHP [2] and SOMANZ [3] recommendations suggest regular follow-up after HDP as well as counselling about women’s individual long-term CVD risk. Designing suitable education for HCPs, appropriate for general use in the Australian healthcare setting and trialling their implementation, would be an important step towards closing the knowledge gap. It is important to establish preferred content and presentation of education for post-HDP health for clinicians, as well as gain insight on enablers and barriers to referral, access and uptake of follow up consultations.

## Conclusion

In our Australian survey of HCP knowledge of risks after HDP, we have found varying knowledge from the targeted professions. Despite ‘high’ knowledge being demonstrated in some areas, significant knowledge gaps were identified. These gaps with regards to general and specific disease risks for women post HDP are important in planning tailored education for HCPs. This may in turn assist in early identification of CVD risk factors in women with a history of HDP and improved subsequent counselling and management.

## Supplementary Information

**Additional file 1.** Long-term health after gestational hypertension or preeclampsia - Health Care Provider Survey. Custom-created survey for the purpose of this study.

**Additional file 2.** Risk factor knowledge by profession and by pregnancy HDP (PE or GH) in numbers and proportions. Shows detailed breakdown of respondent answers, including proportion answering ‘I don’t know’ or skipping questions versus giving a firm but incorrect answer.

## Data Availability

The datasets used and/or analysed during the current study are available from the corresponding author on reasonable request.

## Abbreviations

ACM: the Australian College of Midwives
CH: Chronic hypertension
CSANZ: the Cardiac Society of Australia and New Zealand
CVD: Cardiovascular disease
DRANZCOG: GPs with obstetrics and gynaecology
FRANZCOG: Fellow member of RANZCOG
GH: Gestational hypertension
GP: General practitioner
HCP: Healthcare provider
HDP: Hypertensive disorder of pregnancy
ISSHP: International Society for the Study of Hypertension in Pregnancy
PE: Preeclampsia
RANZCOG: the Royal Australian and New Zealand College of Obstetricians and Gynaecologists
SOMANZ: Society of Obstetric Medicine Australia New Zealand
