# Supplementary material for: Assessing knowledge of healthcare providers concerning cardiovascular risk after hypertensive disorders of pregnancy: an Australian national survey
Source: BMC Pregnancy Childbirth. 2020 Nov 23;20:717. doi: 10.1186/s12884-020-03418-5 (PMC7684922; doi:10.1186/s12884-020-03418-5)
Supplement: Supplementary file 1 — Additional file 1. Long-term health after gestational hypertension or preeclampsia - Health Care Provider Survey. Custom-created survey for the purpose of this study. [file 12884_2020_3418_MOESM1_ESM.pdf]

LONG TERM HEALTH AFTER GESTATIONAL HYPERTENSION OR PREECLAMPSIA - Health Care Provider Survey

Introduction to survey, contact details & Ethics

You are invited to take part in a survey to gain insight into what healthcare providers know about long term health in women who have experienced gestational hypertension or preeclampsia. The study is being conducted by the University of NSW (UNSW), University of Technology Sydney (UTS) and the Sydney Partnership for Health, Education, Research and Enterprise (SPHERE). The study is being undertaken by:

· Dr. Amanda Henry - Senior Lecturer in Obstetrics UNSW & SPHERE member

· Distinguished Professor Caroline Homer - UTS, Midwifery Faculty of Health & SPHERE member

· Dr. Clare Arnott - Cardiologist, Royal Price Alfred Hospital

· Mrs. Heike Roth - PhD Candidate at University of Technology, Sydney

· Mrs. Lynne Roberts - Research Midwife at St George Hospital, SESLHD.

You can complete the survey if you are a currently practicing healthcare provider in Australia. We would like you to reflect on your knowledge and practice over the last three (3) years.

If you are willing to take part in the study, please click on the link and then complete the online survey. This should take about 5-10 minutes to complete and will involve answering questions about you, your current practice and your understanding of long term health in women who have experienced gestational hypertension or preeclampsia.

Participation in this study is entirely voluntary. If you decide to participate and throughout the survey think you would like to withdraw/not complete the survey, you can simply stop and not submit your answers. If you have already submitted your survey, it will not be possible to withdraw the data you have provided as the surveys are anonymous.

The information you provide will not be identifiable and will be kept securely until destroyed as per the South East Sydney Local Health District's requirements.

The results will be published in a research thesis study, in peer reviewed journals and presented at conferences and other professional forums. No one will be able to identify you from this information. If you would like to personally receive results, you will have the option to leave your email details at the end of the survey. The results will be available one (1) year after conclusion of

the survey.

This work is occurring as part of Mrs. Heike Roth's PhD studies and the NHMRC Fellowship of Dr. Henry. Apart from salary support for Dr. Henry, the study is otherwise unfunded.

If you would like any further information about the study please contact the Principal Investigator, Dr. Amanda Henry on 02 91132315 or via email Amanda.henry1@health.nsw.gov.au.

If you experience any distress or concern as a result of completing this survey, please contact Dr. Amanda Henry (details above) or consult your General Practitioner.

If you would like further information about the topic addressed in this study, you can visit the Australian Heart Foundation on the following link: https://www.heartfoundation.org.au/your-heart/women-and-heart-disease/womens-stories.

If you have any concerns or complaints about the conduct of this study, you should contact the Research Support Office of the South Eastern Sydney Local Health District Human Research Ethics Committee which is nominated to receive complaints from research participants. You should contact them on 02 9382 3587, or email SESLHD-RSO@health.nsw.gov.au and quote HREC 18/156.

* 1. I acknowledge that participation in the survey is voluntary.

☐ Enter survey

LONG TERM HEALTH AFTER GESTATIONAL HYPERTENSION OR PREECLAMPSIA - Health Care Provider Survey

ABOUT YOU

These first few questions are to find out about you, your background, and occupation.

2. What is your gender?

- ◯ Female
- ◯ Male
- ◯ Other (please specify)
- ◯ Prefer not to answer

3. Which health professional are you? (Please check the most relevant/current practice answer if more than one apply)

- ◯ General Practitioner
- ◯ Qualified Obstetrician and Gynaecologist, practice gynaecology only
- ◯ Qualified Obstetrician and Gynaecologist, practice obstetrics only
- ◯ Qualified Obstetrician and Gynaecologist, practice both obstetrics & gynaecology
- ◯ Physician with substantial obstetric practice
- ◯ Other (please specify)
- ◯ Cardiologist
- ◯ Physician (other than cardiologist or obstetric physician)
- ◯ Midwife
- ◯ Community/child and family health nurse

4. How long have you been practising in your current profession in Australia?

- ◯ <5 years
- ◯ 5-10 years
- ◯ 11-15 years
- ◯ >15 years
- ◯ Prefer not to answer

5. What state or territory of Australia are you currently practising in?

- ◯ NSW
- ◯ VIC
- ◯ ACT
- ◯ QLD
- ◯ NT
- ◯ SA
- ◯ TAS
- ◯ WA
- ◯ Prefer not to answer

ABOUT YOUR DAILY PRACTICE

These next questions address your daily practice with regards to treating women with a history of gestational hypertension or preeclampsia.

DEFINITIONS:
You may find these useful in order to more easily understand and answer the next questions about the blood pressure conditions addressed in this survey.

Gestational hypertension: the new onset of hypertension (≥140 mmHg systolic or ≥90 mmHg diastolic) after 20 weeks gestation but where the woman is otherwise well (that is, high blood pressure only but no effect on her baby's growth or on her organs).

Preeclampsia: when hypertension (≥140 mmHg systolic or ≥90 mmHg diastolic) is diagnosed after 20 weeks gestation and one or more of the following new-onset conditions are present: (1) Proteinuria (2) Other maternal organ dysfunction such as renal insufficiency, liver involvement, neurological complications, haematological complications (3) Uteroplacental dysfunction and hence potential fetal growth restriction.

6. Do you see/treat women with a history of gestational hypertension or preeclampsia?

- ◯ Yes
- ◯ No
- ◯ I am not sure

ABOUT YOUR DAILY PRACTICE

7. In your usual clinical practice, do you routinely ask women about their pregnancy history including whether they had gestational hypertension or preeclampsia?

- ○ Always
- ○ Often
- ○ Sometimes
- ○ Never

ABOUT YOUR DAILY PRACTICE

8. Do you ever refer women with a history of gestational hypertension and preeclampsia to another health care provider regarding their long-term health because of this specific history?

- Yes
- No
- I am not sure

ABOUT YOUR DAILY PRACTICE

9. Where do you refer women to (e.g. dietician, cardiologist, obstetric or renal physician)? Please write as many as apply.

LONG TERM HEALTH AFTER GESTATIONAL HYPERTENSION OR PREECLAMPSIA - Health Care Provider Survey

LONG-TERM HEALTH RISKS AFTER GESTATIONAL HYPERTENSION OR PREECLAMPSIA

This section looks at comparing the potential long-term health risks for women with a history of gestational hypertension or preeclampsia with women who had no blood pressure complications in pregnancy.

<u>You will be provided with an accurate risk profile at the end of the survey</u>.

10. Do you think that there is an increased risk of developing future cardiovascular disease after gestational hypertension or preeclampsia?

- ◯ Only preeclampsia increases the long-term health risks
- ◯ Only gestational hypertension increases the long-term health risks
- ◯ Both, gestational hypertension and preeclampsia increase the long-term health risks
- ◯ Neither gestational hypertension or preeclampsia increase the long-term health risks
- ◯ I am not sure

11. Compared to a woman who did not have blood pressure problems in pregnancy, how likely is it that women who did have a history of preeclampsia (PE) will develop the following:

	The PE group has a LOWER likelihood of developing it	The PE group has a THE SAME likelihood of developing it	The PE group has a HIGHER likelihood of developing it	I do not know
Chronic hypertension	○	○	○	○
Diabetes	○	○	○	○
Renal disease	○	○	○	○
Breast cancer	○	○	○	○
Cardiac death	○	○	○	○
Ischaemic heart disease/heart attack	○	○	○	○
High blood pressure in another pregnancy	○	○	○	○
Stroke	○	○	○	○
Peripheral vascular disease	○	○	○	○
Leukaemia	○	○	○	○
Seizures	○	○	○	○
Overall mortality risk	○	○	○	○

LONG TERM HEALTH AFTER GESTATIONAL HYPERTENSION OR PREECLAMPSIA - Health Care Provider Survey

12. Compared to a woman who did not have blood pressure problems in pregnancy, how likely is it that women who did have a history of gestational hypertension (GH) will develop the following:

	The GH group has a LOWER likelihood of developing it	The GH group has a THE SAME likelihood of developing it	The GH group has a HIGHER likelihood of developing it	I do not know
Chronic hypertension	○	○	○	○
Diabetes	○	○	○	○
Renal disease	○	○	○	○
Breast cancer	○	○	○	○
Cardiac death	○	○	○	○
Ischaemic heart disease/heart attack	○	○	○	○
High blood pressure in another pregnancy	○	○	○	○
Stroke	○	○	○	○
Peripheral vascular disease	○	○	○	○
Leukaemia	○	○	○	○
Seizures	○	○	○	○
Overall mortality risk	○	○	○	○

13. Compared to a woman who did not have blood pressure problems in pregnancy, how likely is it that women who did have a history of preeclampsia (PE) will develop the following:

	The PE group has a LOWER likelihood of developing it	The PE group has a THE SAME likelihood of developing it	The PE group has a HIGHER likelihood of developing it	I do not know
Chronic hypertension	○	○	○	○
Diabetes	○	○	○	○
Renal disease	○	○	○	○
Breast cancer	○	○	○	○
Cardiac death	○	○	○	○
Ischaemic heart disease/heart attack	○	○	○	○
High blood pressure in another pregnancy	○	○	○	○
Stroke	○	○	○	○
Peripheral vascular disease	○	○	○	○
Leukaemia	○	○	○	○
Seizures	○	○	○	○
Overall mortality risk	○	○	○	○

14. Compared to a woman who did not have blood pressure problems in pregnancy, how likely is it that women who did have a history of gestational hypertension (GH) will develop the following:

	The GH group has a LOWER likelihood of developing it	The GH group has a THE SAME likelihood of developing it	The GH group has a HIGHER likelihood of developing it	I do not know
Chronic hypertension	○	○	○	○
Diabetes	○	○	○	○
Renal disease	○	○	○	○
Breast cancer	○	○	○	○
Cardiac death	○	○	○	○
Ischaemic heart disease/heart attack	○	○	○	○
High blood pressure in another pregnancy	○	○	○	○
Stroke	○	○	○	○
Peripheral vascular disease	○	○	○	○
Leukaemia	○	○	○	○
Seizures	○	○	○	○
Overall mortality risk	○	○	○	○

15. How many years after gestational hypertension or preeclampsia do you think the various signs and symptoms of the above potential risks may start to manifest?

- ◯ < 10 years after pregnancy
- ◯ 10-15 years after pregnancy
- ◯ 16-20 years after pregnancy
- ◯ > 20 years after pregnancy
- ◯ Not sure/don’t know

16. If you have spoken to the women in your care about increased risks of cardiovascular disease after gestational hypertension or preeclampsia, what has been your advice? (please tick all applicable)

- ☐ Assess cardiovascular risk
- ☐ Medication
- ☐ Lifestyle adjustments
- ☐ I have not spoken to women in my care about increased health risks after GH or PE
- ☐ Other (please specify)

17. If you would like to leave any comments about this topic:

LONG TERM HEALTH AFTER GESTATIONAL HYPERTENSION OR PREECLAMPSIA - Health Care Provider Survey

POST-SURVEY OPTIONS AND SUMMARY OF RISK PROFILE

18. **FOCUS GROUP/INTERVIEW OPPORTUNITY-**
(participation certificate provided)

Focus groups (one off - 2hrs max at St George Hospital, Sydney) or more in-depth telephone interviews (one off 30mins max) will be held. The aim is to establish appropriate education content and distribution about long term women's health after hypertensive pregnancy.

If you are interested to participate in either, you can leave your details here.

Name ______________________

Email Address ______________________

Phone Number ______________________

19. Please leave your email address if you wish to receive results from this study (in approx. 1 year). Your details will not be used for any other purpose.

Email Address ______________________

CONTACT
If you experience any distress caused due to the completion of this survey, please contact your GP or the Principal Investigator of this study, Dr Amanda Henry on 02 91132315 or Amanda.henry1@health.nsw.gov.au

For more information on this topic please visit The Australian Heart Foundation on the following link:
https://www.heartfoundation.org.au/your-heart/women-and-heart-disease/womens-stories

RISK PROFILE

There are, unfortunately, some long term health risks associated with having had hypertension in pregnancy.

Women who have had hypertension during pregnancy are about 3 to 4 times more likely to develop **chronic hypertension** than women who did not have a hypertensive pregnancy. They are also about **twice as likely** to get diabetes in later life, even if they did not have diabetes during pregnancy.

Blood pressure diseases are also **more likely** to happen **in the next pregnancy** to women who have already had a previously hypertensive pregnancy compared to women who have not. Therefore, if they have had a hypertensive pregnancy, it is important to be seen early in their next pregnancy. There are treatments that can decrease the chance of recurring problems.

Women are also more likely to get various forms of **cardiovascular disease** (heart disease, stroke, vascular disease) if they have had gestational hypertension or preeclampsia. All of these cardiovascular problems are about **twice as likely** to eventually happen to a woman who has had gestational hypertension or preeclampsia compared to a woman who has not.

Kidney problems are about **5 to 10 times** more common after preeclampsia in particular. Although the **relative risk** of developing renal disease is substantially higher after preeclampsia, the **absolute risk** of long-term renal disease is still low. Unless the woman already had a kidney problem, well over 90% of women after preeclampsia and gestational hypertension **will not have a kidney problem**.

Fortunately, although **seizures** may occur as a result of preeclampsia during pregnancy, women have **no higher** long term risk of risk of seizures compared to women who did not have a complicated pregnancy. There is **no increased risk of getting cancer** (e.g. breast cancer, leukaemia) after having high blood pressure in pregnancy.

For all the long term health **risks**, these **start to go up within 10 years after an affected pregnancy and are ongoing after that.** Therefore, it is recommended that women attend regular blood pressure checks with their GP and discuss any changes they can make to improve their general health. For more general information about heart health and managing health risks, please visit the National Heart Foundation website: https://www.heartfoundation.org.au/your-heart/know-your-risks

THANK YOU FOR YOUR PARTICIPATION
