# Supplementary material for: Assessing knowledge of healthcare providers concerning cardiovascular risk after hypertensive disorders of pregnancy: an Australian national survey
Source: BMC Pregnancy Childbirth. 2020 Nov 23;20:717. doi: 10.1186/s12884-020-03418-5 (PMC7684922; doi:10.1186/s12884-020-03418-5)
Supplement: Supplementary file 2 — Additional file 2. Risk factor knowledge by profession and by pregnancy HDP (PE or GH) in numbers and proportions. Shows detailed breakdown of respondent answers, including proportion answering ‘I don’t know’ or skipping questions versus giving a firm but incorrect answer. [file 12884_2020_3418_MOESM2_ESM.docx]

**Additional file 2 - Risk factor knowledge by profession and by pregnancy HDP (PE or GH) in numbers and proportions**

Chronic hypertension

|  | **Midwives**  **n=171** | | **General Practitioners**  **n=71** | | **Obstetricians**  **n=178** | | **Cardiologists**  **n=26** | |
| --- | --- | --- | --- | --- | --- | --- | --- | --- |
|  | **PE**  **n (%)** | **GH**  **n (%)** | **PE**  **n (%)** | **GH**  **n (%)** | **PE**  **n (%)** | **GH**  **n (%)** | **PE**  **n (%)** | **GH**  **n (%)** |
| Lower likelihood | 0 (0) | 2 (1) | 0 (0) | 0 (0) | 0 (0) | 0 (0) | 0 (0) | 0 (0) |
| Same likelihood | 7 (4) | 12 (7) | 1 (2) | 1 (1) | 3 (2) | 3 (2) | 0 (0) | 0 (0) |
| Higher likelihood | 142 (83) | 132 (77) | 62 (87) | 64 (90) | 166 (93) | 162 (91) | 26 (100) | 26 (100) |
| I do not know | 10 (6) | 6 (4) | 0 (0) | 0 (0) | 0 (0) | 0 (0) | 0 (0) | 0 (0) |
| Did not answer | 12 (7) | 19 (11) | 8 (11) | 6 (8) | 9 (5) | 14 (8) | 0 (0) | 0 (0) |

Diabetes

|  | **Midwives**  **n=171** | | **General Practitioners**  **n=71** | | **Obstetricians**  **n=178** | | **Cardiologists**  **n=26** | |
| --- | --- | --- | --- | --- | --- | --- | --- | --- |
|  | **PE**  **n (%)** | **GH**  **n (%)** | **PE**  **n (%)** | **GH**  **n (%)** | **PE**  **n (%)** | **GH**  **n (%)** | **PE**  **n (%)** | **GH**  **n (%)** |
| Lower likelihood | 1 (1) | 2 (1) | 1 (1) | 0 (0) | 1 (1) | 2 (1) | 0 (0) | 0 (0) |
| Same likelihood | 48 (28) | 54 (32) | 21 (30) | 20 (28) | 68 (38) | 60 (34) | 3 (12) | 5 (19) |
| Higher likelihood | 61 (36) | 51 (30) | 29 (41) | 31 (44) | 76 (43) | 73 (41) | 21 (81) | 17 (65) |
| I do not know | 49 (29) | 45 (26) | 12 (17) | 14 (20) | 22 (12) | 29 (16) | 2 (8) | 4 (15) |
| Did not answer | 12 (7) | 19 (11) | 8 (11) | 6 (8) | 11 (6) | 14 (8) | 0 (0) | 0 (0) |

Renal Disease

|  | **Midwives**  **n=171** | | **General Practitioners**  **n=71** | | **Obstetricians**  **n=178** | | **Cardiologists**  **n=26** | |
| --- | --- | --- | --- | --- | --- | --- | --- | --- |
|  | **PE**  **n (%)** | **GH**  **n (%)** | **PE**  **n (%)** | **GH**  **n (%)** | **PE**  **n (%)** | **GH**  **n (%)** | **PE**  **n (%)** | **GH**  **n (%)** |
| Lower likelihood | 2 (1) | 2 (1) | 0 (0) | 0 (0) | 0 (0) | 1 (1) | 0 (0) | 0 (0) |
| Same likelihood | 9 (5) | 27 (16) | 1 (1) | 9 (13) | 11 (6) | 20 (11) | 0 (0) | 1 (4) |
| Higher likelihood | 128 (75) | 102 (60) | 61 (86) | 53 (75) | 152 (85) | 133 (75) | 26 (100) | 23 (89) |
| I do not know | 20 (12) | 20 (12) | 1 (1) | 3 (4) | 5 (3) | 11 (6) | 0 (0) | 2 (8) |
| Did not answer | 12 (7) | 20 (12) | 8 (11) | 6 (8) | 10 (6) | 13 (7) | 0 (0) | 0 (0) |

Cardiac Death

|  | **Midwives**  **n=171** | | **General Practitioners**  **n=71** | | **Obstetricians**  **n=178** | | **Cardiologists**  **n=26** | |
| --- | --- | --- | --- | --- | --- | --- | --- | --- |
|  | **PE**  **n (%)** | **GH**  **n (%)** | **PE**  **n (%)** | **GH**  **n (%)** | **PE**  **n (%)** | **GH**  **n (%)** | **PE**  **n (%)** | **GH**  **n (%)** |
| Lower likelihood | 1 (1) | 3 (2) | 0 (0) | 0 (0) | 0 (0) | 1 (1) | 0 (0) | 0 (0) |
| Same likelihood | 13 (8) | 20 (12) | 1 (1) | 8 (11) | 4 (2) | 17 (10) | 1 (4) | 3 (12) |
| Higher likelihood | 110 (65) | 93 (54) | 57 (80) | 50 (70) | 149 (84) | 130 (73) | 23 (89) | 21 (81) |
| I do not know | 34 (20) | 35 (20) | 5 (7) | 7 (10) | 14 (8) | 17 (10) | 2 (7) | 2 (7) |
| Did not answer | 13 (8) | 20 (12) | 8 (11) | 6 (8) | 11 (6) | 13 (7) | 0 (0) | 0 (0) |

Ischaemic Heart Disease/Heart attack

|  | **Midwives**  **n=171** | | **General Practitioners**  **n=71** | | **Obstetricians**  **n=178** | | **Cardiologists**  **n=26** | |
| --- | --- | --- | --- | --- | --- | --- | --- | --- |
|  | **PE**  **n (%)** | **GH**  **n (%)** | **PE**  **n (%)** | **GH**  **n (%)** | **PE**  **n (%)** | **GH**  **n (%)** | **PE**  **n (%)** | **GH**  **n (%)** |
| Lower likelihood | 1 (1) | 3 (2) | 0 (0) | 0 (0) | 0 (0) | 1 (1) | 0 (0) | 0 (0) |
| Same likelihood | 16 (9) | 21 (12) | 1 (1) | 4 (8) | 8 (4) | 15 (8) | 1 (4) | 1 (4) |
| Higher likelihood | 113 (66) | 97 (57) | 57 (80) | 58 (82) | 155 (87) | 140 (79) | 25 (96) | 24 (92) |
| I do not know | 29 (17) | 30 (18) | 5 (7) | 3 (4) | 6 (3) | 9 (5) | 0 (0) | 1 (4) |
| Did not answer | 12 (7) | 20 (12) | 8 (11) | 6 (8) | 9 (5) | 13 (7) | 0 (0) | 0 (0) |

HDP next pregnancy

|  | **Midwives**  **n=171** | | **General Practitioners**  **n=71** | | **Obstetricians**  **n=178** | | **Cardiologists**  **n=26** | |
| --- | --- | --- | --- | --- | --- | --- | --- | --- |
|  | **PE**  **n (%)** | **GH**  **n (%)** | **PE**  **n (%)** | **GH**  **n (%)** | **PE**  **n (%)** | **GH**  **n (%)** | **PE**  **n (%)** | **GH**  **n (%)** |
| Lower likelihood | 0 (0) | 3 (2) | 0 (0) | 0 (0) | 0 (0) | 1 (1) | 0 (0) | 0 (0) |
| Same likelihood | 12 (7) | 20 (12) | 0 (0) | 4 (6) | 0 (0) | 15 (8) | 1 (4) | 1(4) |
| Higher likelihood | 146 (85) | 100 (58) | 63 (89) | 58 (82) | 169 (94) | 140 (79) | 25 (96) | 24 (92) |
| I do not know | 1 (1) | 29 (17) | 0 (0) | 3 (4) | 0 (0) | 9 (5) | 0 (0) | 1 (4) |
| Did not answer | 12 (7) | 19 (11) | 8 (11) | 6 (8) | 9 (5) | 13 (7) | 0 (0) | 0 (0) |

Stroke

|  | **Midwives**  **n=171** | | **General Practitioners**  **n=71** | | **Obstetricians**  **n=178** | | **Cardiologists**  **n=26** | |
| --- | --- | --- | --- | --- | --- | --- | --- | --- |
|  | **PE**  **n (%)** | **GH**  **n (%)** | **PE**  **n (%)** | **GH**  **n (%)** | **PE**  **n (%)** | **GH**  **n (%)** | **PE**  **n (%)** | **GH**  **n (%)** |
| Lower likelihood | 1 (1) | 3 (2) | 0 (0) | 0 (0) | 0 (0) | 1 (1) | 0 (0) | 0 (0) |
| Same likelihood | 17 (10) | 19 (11) | 1 (1) | 6 (8) | 12 (7) | 22 (12) | 1 (4) | 1 (4) |
| Higher likelihood | 121 (71) | 105 (61) | 57 (80) | 54 (76) | 150 (84) | 128 (72) | 24 (92) | 23 (89) |
| I do not know | 20 (12) | 24 (14) | 5 (7) | 5 (7) | 7 (4) | 13 (7) | 1 (4) | 2 (8) |
| Did not answer | 12 (7) | 20 (12) | 8 (11) | 6 (8) | 9 (5) | 14 (8) | 0 (0) | 0 (0) |

Peripheral Vascular Disease

|  | **Midwives**  **n=171** | | **General Practitioners**  **n=71** | | **Obstetricians**  **n=178** | | **Cardiologists**  **n=26** | |
| --- | --- | --- | --- | --- | --- | --- | --- | --- |
|  | **PE**  **n (%)** | **GH**  **n (%)** | **PE**  **n (%)** | **GH**  **n (%)** | **PE**  **n (%)** | **GH**  **n (%)** | **PE**  **n (%)** | **GH**  **n (%)** |
| Lower likelihood | 1 (1) | 3 (2) | 0 (0) | 0 (0) | 0 (0) | 1 (1) | 0 (0) | 0 (0) |
| Same likelihood | 23 (13) | 21 (12) | 3 (4) | 9 (13) | 25 (14) | 29 (16) | 3 (12) | 4 (15) |
| Higher likelihood | 96 (56) | 84 (49) | 44 (62) | 38 (54) | 109 (61) | 100 (56) | 19 (73) | 17 (65) |
| I do not know | 37 (22) | 44 (26) | 16 (23) | 17 (24) | 37 (21) | 32 (18) | 4 (15) | 5 (19) |
| Did not answer | 14 (8) | 19 (11) | 8 (11) | 7 (10) | 7 (4) | 16 (9) | 0 (0) | 0 (0) |

Overall Mortality

|  | **Midwives**  **n=171** | | **General Practitioners**  **n=71** | | **Obstetricians**  **n=178** | | **Cardiologists**  **n=26** | |
| --- | --- | --- | --- | --- | --- | --- | --- | --- |
|  | **PE**  **n (%)** | **GH**  **n (%)** | **PE**  **n (%)** | **GH**  **n (%)** | **PE**  **n (%)** | **GH**  **n (%)** | **PE**  **n (%)** | **GH**  **n (%)** |
| Lower likelihood | 1 (1) | 2 (1) | 0 (0) | 0 (0) | 0 (0) | 1 (1) | 0 (0) | 0 (0) |
| Same likelihood | 11 (6) | 16 (9) | 0 (0) | 10 (14) | 7 (4) | 13 (7) | 0 (0) | 2 (8) |
| Higher likelihood | 124 (73) | 104 (61) | 58 (82) | 50 (70) | 147 (83) | 133 (75) | 24 (92) | 23 (89) |
| I do not know | 23 (13) | 28 (16) | 5 (7) | 4 (6) | 15 (8) | 18 (10) | 2 (8) | 1 (4) |
| Did not answer | 12 (7) | 21 (12) | 8 (11) | 7 (10) | 9 (5) | 13 (7) | 0 (0) | 0 (0) |

Supplementary Table 10: Breast Cancer

|  | **Midwives**  **n=171** | | **General Practitioners**  **n=71** | | **Obstetricians**  **n=178** | | **Cardiologists**  **n=26** | |
| --- | --- | --- | --- | --- | --- | --- | --- | --- |
|  | **PE**  **n (%)** | **GH**  **n (%)** | **PE**  **n (%)** | **GH**  **n (%)** | **PE**  **n (%)** | **GH**  **n (%)** | **PE**  **n (%)** | **GH**  **n (%)** |
| Lower likelihood | 4 (2) | 4 (2) | 0 (0) | 0 (0) | 4 (2) | 3 (2) | 0 (0) | 0 (0) |
| Same likelihood | 56 (33) | 56 (33) | 32 (45) | 31 (44) | 90 (51) | 87 (49) | 11 (42) | 13 (50) |
| Higher likelihood | 5 (3) | 5 (3) | 0 (0) | 2 (3) | 1 (1) | 5 (3) | 3 (12) | 2 (8) |
| I do not know | 94 (55) | 86 (50) | 31 (44) | 32 (45) | 73 (41) | 69 (39) | 12 (46) | 11 (42) |
| Did not answer | 12 (7) | 20 (12) | 8 (11) | 6 (8) | 10 (6) | 14 (8) | 0 (0) | 0 (0) |

Supplementary Table 11: Leukaemia

|  | **Midwives**  **n=171** | | **General Practitioners**  **n=71** | | **Obstetricians**  **n=178** | | **Cardiologists**  **n=26** | |
| --- | --- | --- | --- | --- | --- | --- | --- | --- |
|  | **PE**  **n (%)** | **GH**  **n (%)** | **PE**  **n (%)** | **GH**  **n (%)** | **PE**  **n (%)** | **GH**  **n (%)** | **PE**  **n (%)** | **GH**  **n (%)** |
| Lower likelihood | 2 (1) | 3 (2) | 1 (1) | 0 (0) | 2 (1) | 2 (1) | 0 (0) | 0 (0) |
| Same likelihood | 58 (34) | 56 (33) | 24 (34) | 28 (39) | 86 (48) | 86 (48) | 12 (46) | 13 (50) |
| Higher likelihood | 3 (2) | 3 (2) | 0 (0) | 0 (0) | 1 (1) | 1 (1) | 1 (4) | 1 (4) |
| I do not know | 96 (56) | 90 (53) | 38 (54) | 35 (49) | 79 (44) | 74 (42) | 13 (50) | 12 (46) |
| Did not answer | 12 (7) | 19 (11) | 8 (11) | 8 (11) | 10 (6) | 15 (8) | 0 (0) | 0 (0) |

Supplementary Table 12: Seizures

|  | **Midwives**  **n=171** | | **General Practitioners**  **n=71** | | **Obstetricians**  **n=178** | | **Cardiologists**  **n=26** | |
| --- | --- | --- | --- | --- | --- | --- | --- | --- |
|  | **PE**  **n (%)** | **GH**  **n (%)** | **PE**  **n (%)** | **GH**  **n (%)** | **PE**  **n (%)** | **GH**  **n (%)** | **PE**  **n (%)** | **GH**  **n (%)** |
| Lower likelihood | 1 (1) | 2 (1) | 0 (0) | 0 (0) | 3 (2) | 3 (2) | 0 (0) | 0 (0) |
| Same likelihood | 33 (19) | 51 (30) | 25 (35) | 37 (52) | 83 (47) | 86 (48) | 7 (27) | 9 (35) |
| Higher likelihood | 49 (29) | 28 (16) | 12 (17) | 5 (7) | 20 (11) | 10 (6) | 9 (35) | 7 (27) |
| I do not know | 74 (43) | 70 (41) | 26 (37) | 23 (32) | 62 (35) | 64 (36) | 10 (39) | 9 (35) |
| Did not answer | 14 (8) | 20 (12) | 8 (11) | 6 (8) | 10 (5) | 15 (8) | 0 (0) | 1 (4) |
